# Epidemiology and patients’ self-reported knowledge of implantable medical devices: Results of a cross-sectional survey in Hungary

**DOI:** 10.1371/journal.pone.0284577

**Published:** 2023-04-18

**Authors:** Áron Hölgyesi, Barbara Tóth, Miklós Kozlovszky, József Kuti, Miklós Weszl, György Balázs, Petra Baji, Levente Kovács, László Gulácsi, Zsombor Zrubka, Márta Péntek

**Affiliations:** 1 Doctoral School of Molecular Medicine, Semmelweis University, Budapest, Hungary; 2 Doctoral School of Applied Informatics and Applied Mathemathics, Óbuda University, Budapest, Hungary; 3 BioTech Research Center, Óbuda University, Budapest, Hungary; 4 Antal Bejczy Center for Intelligent Robotics, Óbuda University, Budapest, Hungary; 5 Department of Translational Medicine, Semmelweis University, Budapest, Hungary; 6 EMKI-CERT Ltd., Budapest, Hungary; 7 Department of Health Economics, Corvinus University of Budapest, Budapest, Hungary; 8 Musculoskeletal Research Unit, University of Bristol, Bristol, United Kingdom; 9 Physiological Controls Research Center, University Research and Innovation Center, Óbuda University, Budapest, Hungary; 10 Health Economics Research Center, University Research and Innovation Center, Óbuda University, Budapest, Hungary; 11 Corvinus Institute of Advanced Studies, Corvinus University of Budapest, Budapest, Hungary; Saarland University, GERMANY

## Abstract

**Background:**

Implantable medical devices (IMDs) are medical instruments embedded inside the body. Well-informed and empowered patients living with IMDs are key players of improving IMD-related patient safety and health outcomes. However, little is known about IMD patients’ epidemiology, characteristics, and current awareness levels. Our primary aim was to investigate the point and lifetime prevalence of patients living with IMDs. Patients’ IMD-related knowledge and determinants of IMDs’ impact on their life were also explored.

**Methods:**

An online cross-sectional survey was conducted. Respondents’ IMD history, whether they received instructions for use and IMD’s overall impact on life were recorded by self-reports. Patients’ knowledge about living with IMDs was assessed on visual analogue scales (VAS, 0–10). Shared decision-making was analyzed by the 9-item Shared Decision Making Questionnaire (SDM-Q-9). Descriptive statistics and subgroup comparisons between IMD wearers were performed for statistical differences. Significant determinants of IMD’s overall impact on life were examined in linear regression analysis.

**Results:**

In the total sample (N = 1400, mean age 58.1 ±11.1; female 53.7%), nearly one third of respondents were living with IMD (30.9%; 433/1400). Among them, the most frequent IMDs were tooth implants (30.9%) and intraocular lens (26.8%). Mean knowledge VAS scores were similar (range: 5.5 ±3.8–6.5 ±3.2) but differences by IMD types were observed. Patients who received instructions for use or reported better impact on life indicated higher self-reported knowledge. Regression confirmed that patients’ knowledge was significant predictor of IMD’s impact on life, but this effect was overwritten by the SDM-Q-9.

**Conclusions:**

This first comprehensive epidemiological study on IMDs provides basic data for public health strategy planning alongside the implementation of MDR. Improved self-perceived outcomes were associated with higher knowledge hence education of patients receiving IMD deserves consideration. We suggest to investigate further the role of shared decision-making on IMD’s overall impact on patients’ life in future prospective studies.

## Introduction

The new medical device regulation (MDR) has been applied since May 2021 in the European Union (EU 2017/745), aiming to guarantee high level of protection of health for patients and to improve health outcomes by setting high common quality and safety standards for medical devices [1]. To reach these goals, empowerment of patients in the use and decision-making related to the device throughout its lifetime is essential. Hence manufacturers are imposed to provide sufficient information for the users of medical devices in a transparent and appropriate way. The term implantable medical devices (IMDs) refers to a special group of devices that are introduced in the body either totally for permanent use or partially to remain their for at least 30 days [1]. IMDs are subject to specific rules in the MDR. One of these rules is that manufacturers should provide an implant card together with the IMD, bearing basic information that allows device level identification, describes the measures to be taken by the patient in case of external influences and enables users to recognise safety-related issues. Furthermore, relevant information should be provided in a way that grants quick access for all patients, with making updates available on the website of the manufacturer which in itself implies certain level of internet use and eHealth literacy among patients.

To understand the impact of the new regulation from the patients’ perspectives, the challenges of their involvement in the proccess and their capability to collaborate, as well as the potential need for public health interventions in this new context, basic descriptive epidemiological data on the affected people and an insight into patients’ current knowledge regarding their own IMD are needed. Available studies concerning the prevalence of IMDs are restricted mainly to single-center observations and IMD-specific registries [2, 3]. Moreover, patients’ knowledge and its association with health outcomes have only been studied in particular IMD groups, however, comprehensive assessments covering broad spectrum of IMDs are lacking [4–7].

Most IMDs differ with respect to their indication, handling, probability of malfunction and associated risk factors. These differences suggest that the level of knowledge and its impact on patients’ life varies by IMD type. However, less is known about the association of patient-related factors and outcomes of IMDs. There are some features that presumably have an influence regardless of the tpye and indication of IMD (e.g.: sociodemographic characteristics of the patient). Nonetheless, other modifiable factors might also play an important role, such as access to and interiorisation of proper information regarding the use of the IMD, the level of digital health literacy or the degree of patient involvement in IMD-related medical decision-making [8–10]. Specific tools have been developed to assess most of these factors, making it possible to analyse their association with IMD-related health knowledge and outcome [11–13].

The aim of our study was to assess the prevalence of IMDs in the Hungarian population, to examine patients’ IMD-related knowledge and to identify relevant determinants of IMD’s overall impact on patients’ life.

## Methods

### Data collection

An online, cross-sectional, web-based survey study was carried out in July 2021, involving a sample of the Hungarian general population aged 40 years and over. To ensure representativeness, quota sampling was used with quotas for sex, age, education and residency based on the 2011 Population Census in Hungary [14]. In order to achieve representativeness according to these criterias, the usually needed sample size is N = 1000. Furthermore, as the prevalence of IMDs was assumed to be 35% and the confidence interval was set at 95%, the required number of respondents was calculated to be 1398. A survey company was responsible for the recruitment and data collection, for which participants were reached through an online access panel by e-mail. Data on the completion rate were confidential. Ethical approval was obtained from the Hungarian Medical Research Council (no. IV/5651-1/2021/EKU). Respondents were informed that participation in the survey was voluntary, their data would remain anonymous, impersonal and would be used solely for scientific purposes. Respondents needed to provide their written informed consent online before the start of the survey. The survey company was also responsible for compliance with the GDPR and data protection.

### The questionnaire

This study was part of a larger survey comprising three research modules: 1.) epidemiology and knowledge about IMDs, including involvement in decision-making about IMD implantation; 2.) Subjective preferences regarding artificial intelligence-based medical devices 3.) subjective expectations regarding having IMDs at older ages. In this paper we report the results of the first research module.

The following sociodemographic variables were surveyed: sex (female or male), age, educational level (primary, secondary or tertiary), place of residency (living in a city, village or in the capital), employment status (having a paid job) and family status (married or having a partnership; living with someone in the household). The monthly net income of the household was questioned in 11 range categories, then per capita net income was calculated by dividing the midrange value of categories by the number of household members, without adjustment for the number of children. The method proposed by Parker and Fenwick was used to estimate the midrange value of the upper category [15]. Quintiles of monthly net household income per capita was used to form income groups. The second, third, fourth and fifth quintiles were calculated from the means of the third to eighth national decile groups provided by the Hungarian Central Statistical Office [16]. Respondents could complete the survey only once, were able to review and change their answers. and received no reward for their participation. In the final analysis, only fully completed surveys were included. The survey was created and developed by our research team. No preliminary testing was performed. Participants’ health status, digital health literacy and involvement in medical decision-making about the IMD were surveyed by standard validated outcome measures (detailed below).

### Epidemiology of implantable medical devices (IMD)

Respondents were provided with the following list of IMDs: hip implant, knee implant, spine implant, intraocular lens, bone fixation, dental implant, dental bone graft (for dental implants), pacemaker, artificial heart valve, coronary stent, abdominal mesh, breast implant, glucose sensor and intrauterin device (for females) [17]. Other devices could have been specified as ‘other implants’. Self-reported lifetime prevalence (ever received) and point-prevalence (still in) of IMDs, as well as respondents’ age at implantation were recorded. Duration of living with IMD was calculated for those currently living with IMD.

Lifetime and point-prevalence of IMDs were assessed by age groups and sex. For the analyses, some of the IMD types were combined into greater categories (dental implant and dental bone graft into ‘Dental implants’; pacemaker, artificial heart valve and coronary stent into ‘Cardiovascular impants’; knee implant, hip implant and spine implant into ‘Orthopedic implants’) to ensure better understanding of the results.

### Patients’ currently living with IMD: Knowledge and IMD’s overall impact on their life

Those who were currently living with IMDs were asked whether they received written ‘Instructions for use’ for the IMD. The four response options were ‘yes, and read it’, ‘yes, but did not read it’, ‘no’ and ‘do not remember’. Patients were asked to indicate their self-perceived familiarity with general instructions for use (‘How familiar are you with the instructions and lifestyle advice for the daily use of the implanted device?’) and safety requirements (‘How familiar are you with the specific safety requirements for the implanted device you are using?’), as well as their self-perceived ability to recognise need for medical control (‘How confident are you that you will be able to recognise in time if there is a problem with the implanted device that needs medical control?’) and for information security or privacy control (‘How confident are you that you will be able to recognise in time if there is a problem with the implanted device that requires information security or privacy control?’). The latter question was given only to those who were wearing electronic devices (pacemaker or glucose sensor). A visual analogue scale (VAS) was provided for each question, where 0 represented the worst and 10 represented the best possible familiarity (‘not at all’ and ‘completely’, respectively).

Patients’ subjective opinion on the IMD’s overall impact on their life (hereinafter ‘IMD’s overall impact’) was measured by a single question (‘How do you think your IMD affected your life overall? Including the circumstances of the implantation and the IMDs’ impact on your quality of life and daily activities.’), with a 5-level response scale (1 - ‘very negative’; 5 - ‘very positive’).

### Outcome measurement tools

#### EQ-5D-5L

The EQ-5D-5L, developed by the EuroQol Group, is a generic health status measure that evaluates health-related quality of life in the following five health domains: mobility, self-care, usual activities, pain/discomfort, anxiety/depression. For each domain, there are five statements representing different problem levels in the given domain (1 –‘no problems’, 2 –‘slight problems’, 3 –‘moderate problems’, 4 –‘severe problems’, 5 –‘unable to’/‘extreme’). Respondents are asked to indicate in each domain the one that best describes their health on that day. Hungarian tariffs were used to calculate the EQ-5D-5L index scores (range: -0.848–1.000) [18]. An additional item, the EQ VAS measures respondents self-reported health. The EQ VAS ranges from 0 to 100, where 0 and 100 represents the worst and best possible health states that the respondent can imagine [19].

#### Electronic Health Literacy Scale (eHEALS)

The eHeals was developed to measure respondents’ confidence, knowledge and ability to find, understand and apply health related electronic information [20]. The scale comprises 8 items, with one question per item, that covers 4 domains of digital health literacy: awareness (items 1 and 2), search for information (items 3 and 4), appraisal of health resources (items 6 and 7), and utilisation of health information (items 5 and 8). Responses are scored on a 5 level Likert-scale (1 - ‘strongly disagree’; 5 - ‘strongly agree’). Item scores are added up to calculate the total score (ranging form 8 to 40), higher score indicate better eHealth literacy. There are two additional questions to assess the respondents’ interest in using the Internet to find health information that are not considered in the final score. In our analysis, the validated Hungarian version was used [11].

#### Shared Decision Making Questionnaire (SDM-Q-9)

The SDM-Q-9 was designed to assess the extent to which patients are involved in decision-making regarding their own health [21]. It comprises two open-ended questions (recent health problem that was consulted with a healthcare provider and the decision that was made) followed by nine statements concerning different aspects of the decision-making process and the communication with the physician. Each statement can be rated on a six point scale (0 - ‘completely disagree’; 5 - ‘completely agree’). The total score (ranging from 0 to 45) is calculated by summing up the individual scores given for each item, higher scores indicate greater level of self-perceived involvement in decision-making. In our analysis, the recently developed Hungarian language version of the tool was used [13].

### Statistical analysis

Socio-demographic characteristics, health status and occurence of IMDs were analyzed via descriptive statistical methods. All statistical analysis, including the assessment of the prevalence of IMDs were done on the unweighted sample. Differences in mean age at implantation and duration of living with IMD were examined with analysis of variance (ANOVA).

Patients’ knowledge was analysed by socio-demographic subgroups, health state (EQ-5D-5L), whether instructions for use have been received and IMD’s overall impact on life. Between group differences were compared with Kruskal Wallis tests. The associations between patient’s knowledge (VAS scales), health state (EQ-5D-5L, EQ VAS), electronic health literacy (eHEALS) and shared decision-making (SDM-Q-9) were examined with Spearman’s rank correlation (> 0.5 –strong, 0.5–0.3 –moderate, < 0.3 –weak) [22]. The mean of the knowledge VAS scales was calculated to represent patients’ general knowledge about IMDs. To test the consistency of this combined knowledge score, Cronbach’s alfa was calculated (0.7–0.8: acceptable, 0.8–0.9: good, > 0.9: excellent) [23]. Patients’ knowledge was assessed by IMD type and sex; differences between men and women (by the combined knowledge score) were analysed in a linear regression model in which all IMD types and their interaction with sex were included as predictors.

Responses on the received instrucions for use question and the IMD’s overall impact on life were analysed by socio-demographic subgroups and by the type of IMD. Multiple linear regression was carried out to find relevant determinants of IMD’s overall impact on life. We examined the extent to which patients’ knowledge can predict IMD’s overall impact on life, and whether having received instructions for use, digital health literacy (eHEALS) and level of shared decision-making (SDM-Q-9) modify this effect if adjustments are applied for IMD type, sociodemographics, the duration since the first implant and health state. Seven models were developed. As a base case, all IMD types were added together to the first model and further variables were added one by one to ensure sufficient predictor selection. Received instructions for use was included as a dummy variable, taking value 1 for those who had have received instructions and read it, and taking value 0 for other response options. The significance of each predictors was determined based on the results of the t-statistics. Multicollinearity was tested by measuring the variance inflation factor (VIF).

Cases in which respondents indicated that they do not wish to or could not answer a certain question were considered as missing values. Missing data were excluded from the analysis. The analysis was performed using Stata version 17 statistical software (StataCorp LCC., College Station, TX, USA).

## Results

### Sample

All in all, 1,400 respondents who have fully completed the survey were included in the study. The mean age was 58.3 (± 11.1) years. The sample was comparable with the general 40+ years old Hungarian population with respect to sex, age and residency, but educational level was slightly higher. The mean EQ-5D-5L, EQ VAS, and eHEALS were 0.83 (±0.26), 75.1 (±19.9) and 28.1 (±5.8), respectively. Main characteristics of the sample are summarised in Table 1.

**Table 1:** Sociodemographic characteristics of the sample.

	Total sample		Subsample living with IMD		Microcensus 2016
Variables	N	%	N	%	% of 40+ general population
**Total**	1400	100	433	100	
**Sex**					
*Men*	648	46.3	213	49.2	44.7
*Women*	752	53.7	220	50.8	55.3
**Age**					
*40–44*	190	13.6	40	9.2	15.3
*45–49*	188	13.4	40	9.2	13.4
*50–54*	163	11.6	41	9.5	11.1
*55–59*	198	14.1	49	11.3	11.9
*60–64*	227	16.2	73	16.9	13.8
*65–69*	182	13.0	66	15.2	11.2
*70–74*	127	9.1	57	13.2	8.6
*75+*	125	8.9	67	15.5	14.6
**Education**					
*Primary*	410	29.3	104	24.0	50.0
*Secondary*	533	38.1	153	35.3	29.8
*Tertiary*	457	32.6	176	40.6	20.2
**Settlement type**					
*Capital*	315	22.5	117	27.0	18.0*
*Town*	749	53.5	234	54.0	52.7*
*Village*	336	24.0	82	18.9	29.3*
**Married/having a partner**					
*Yes*	854	61.0	258	59.6	64.9
*No*	546	39.0	175	40.4	35.1
**Living with someone in the household**					
*Yes*	1064	76.0	319	73.7	-
*No*	336	24.0	114	26.3	-
**Paid work**					
*Yes*	683	48.8	171	39.5	48.9
*No*	717	51.2	262	60.5	51.1
**Houshold income category (per capita)** ^**a**^					
*1st quintile*	261	18.6	60	13.9	-
*2nd quintile*	224	16.0	76	17.6	-
*3rd quintile*	237	16.9	71	16.4	-
*4th quintile*	201	14.4	63	14.5	-
*5th quintile*	260	18.6	107	24.7	-
*Missing*	217	15.5	56	12.9	-

^a^Conversion: 1 EUR = 330.73 HUF (the exchange rate in October 2021)

*Data represents the distribution in the general population regardless of age

### Epidemiology of IMDs

Altogether 41.7% (584/1400) of the sample have had at least one IMD ever. The proportion of participants who have had one, two, three or more implants in their life was 29.4%, 9.6%, 2.3% and 0.4%, respectively. IMDs with the highest lifetime prevalence were bone fixation (12.3%), tooth implant (10.1%), intrauterine device (9.4%) and intraocular lens (8.5%) (Table 2). Furthermore, 29 respondents indicated ‘other’ IMDs that were not included in the prespecified list (e.g., hand and wrist implants N = 5; stents N = 4; shoulder implant N = 3).

**Table 2:** Prevalence and duration of implantable medical devices (IMD).

	Participants who have ever had IMD, N = 583		Age at implantation (ever had)		Subsample currently living with IMD, N = 433		Duration of IMD in the subsample living with IMD	
	N	% of total sample	Mean (years)	SD	N	% of subsample	Mean (years)	SD
Bone fixation (e.g. plate, screw)	172	12.3	40.5	16.4	77	17.8	11.5	10.5
Tooth implant	141	10.1	51.5	13.4	134	30.9	8.4	7.9
Intrauterin device	131	9.4	32.9	8	18	4.2	9.2	10.4
Cataract surgery (artificial lens in the eye)	119	8.5	61.3	12.7	116	26.8	6.8	7.1
Abdominal surgical mesh	38	2.7	55.4	11.9	37	8.5	8.4	6
Bone graft for dental implant	35	2.5	51.5	12.3	32	7.4	7.2	6.2
Hip replacement	33	2.4	58.3	10.8	32	7.4	8.4	7.7
Other type	29	2.1	49.2	14.8	19	4.4	11.1	8.6
Coronary stent	27	1.9	59.4	10.5	26	6.0	7.6	5.6
Knee replacement	19	1.4	55.3	10.3	19	4.4	7.2	6
Spinal implant	19	1.4	53.4	14.1	18	4.2	10.1	7.9
Pacemaker	15	1.1	53.9	17.9	15	3.5	8.8	7
Breast implant	13	0.9	42.5	8.7	13	3.0	12.6	10.3
Artificial heart valve	6	0.4	48.2	20.4	6	1.4	14.3	11.3
Glucose sensor	4	0.3	31.8	24.3	1	0.2	13	-

In the total sample, mean age at first implantation was 45.2 (±16.2) years and it varied significantly (ANOVA F_(14,548)_ = 7.74, p <0.001) by IMD type. Glucose sensor, intrauterine device, bone fixation and breast implant were operated at the lowest ages, while coronary stent and intraocular lens at the highest ages.

#### Lifetime prevalence of IMDs by age group and sex

**Fig 1:**
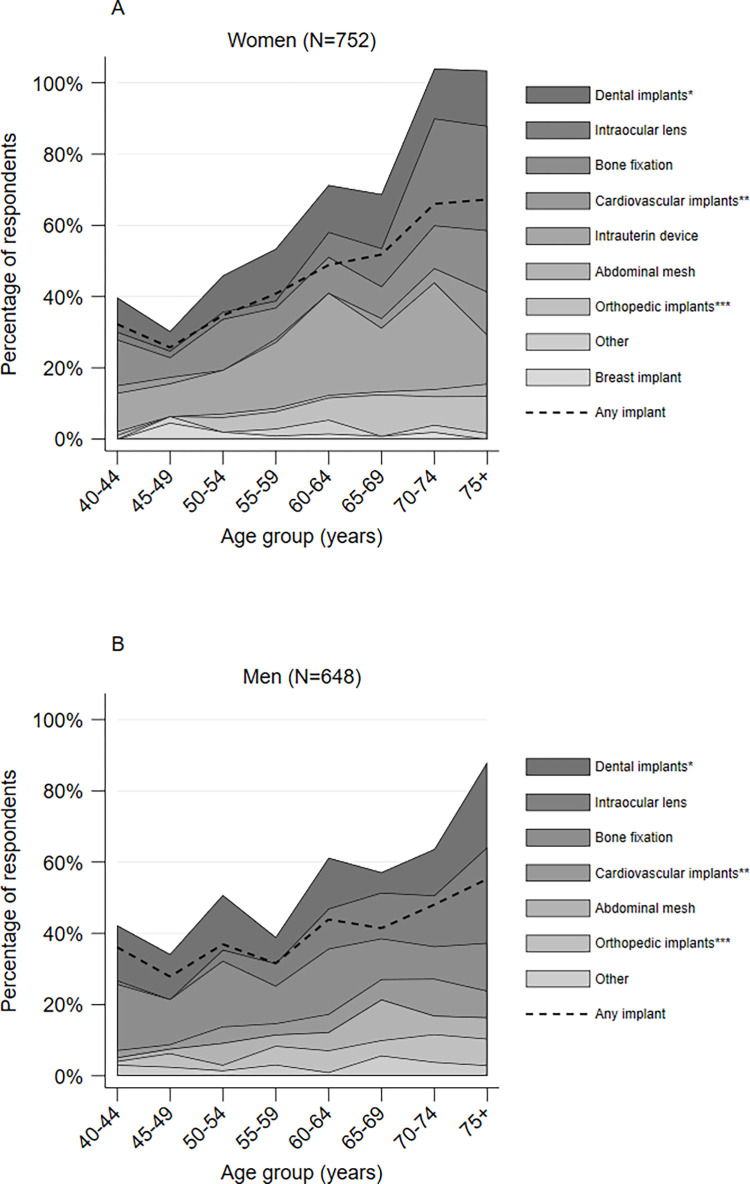
Distribution of ever received IMDs in the total sample by age groups and sex. *Dental implants inculde tooth implant and dental bone graft; **Cardiovascular implants include pacemaker, artificial heart valve and coronary stent; ***Orthopedic implants include hip, knee and spine implants; Data for the category ‘glucose sensor’ are not presented as only four women cases were identified. Similarly, the category ‘breast implant’ is not included for men since only one case was found.

Distribution of IMD types by age groups and sex are presented in Fig 1A and 1B. Overall amount of IMDs and the proportion of respondents who have ever had any implant increased with age. In women, the lifetime prevalence of dental implants and bone fixation was relatively balanced across age groups, while the lifetime prevalence of other IMDs showed an age related tendency (with greater prevalence in older ages). Similar pattern can be observed in men, however, bone fixation tended to be slightly more common in younger age groups.

#### Point prevalence of IMDs

All in all, 30.9% (433/1400) of the sample were living with IMDs at the time of the survey. Among them 74.6%, 21.0%, 4.2% and 0.2% were having one, two, three or more different IMDs, respectively. The only occuring electronic IMDs were pacemakers and glucose sensors. The highest point prevalences among respondents living with IMDs were recorded for tooth implant (30.9%), intraocular lens (26.8%), and bone fixation (17.8%). The longest duration of living with IMD was recorded for artificial heart valve and breast implant. The point prevalence of IMDs, mean age at implantation and duration of living with the device are summarized in Table 2.

### Patients’ knowledge regarding living with IMDs

**Fig 2:**
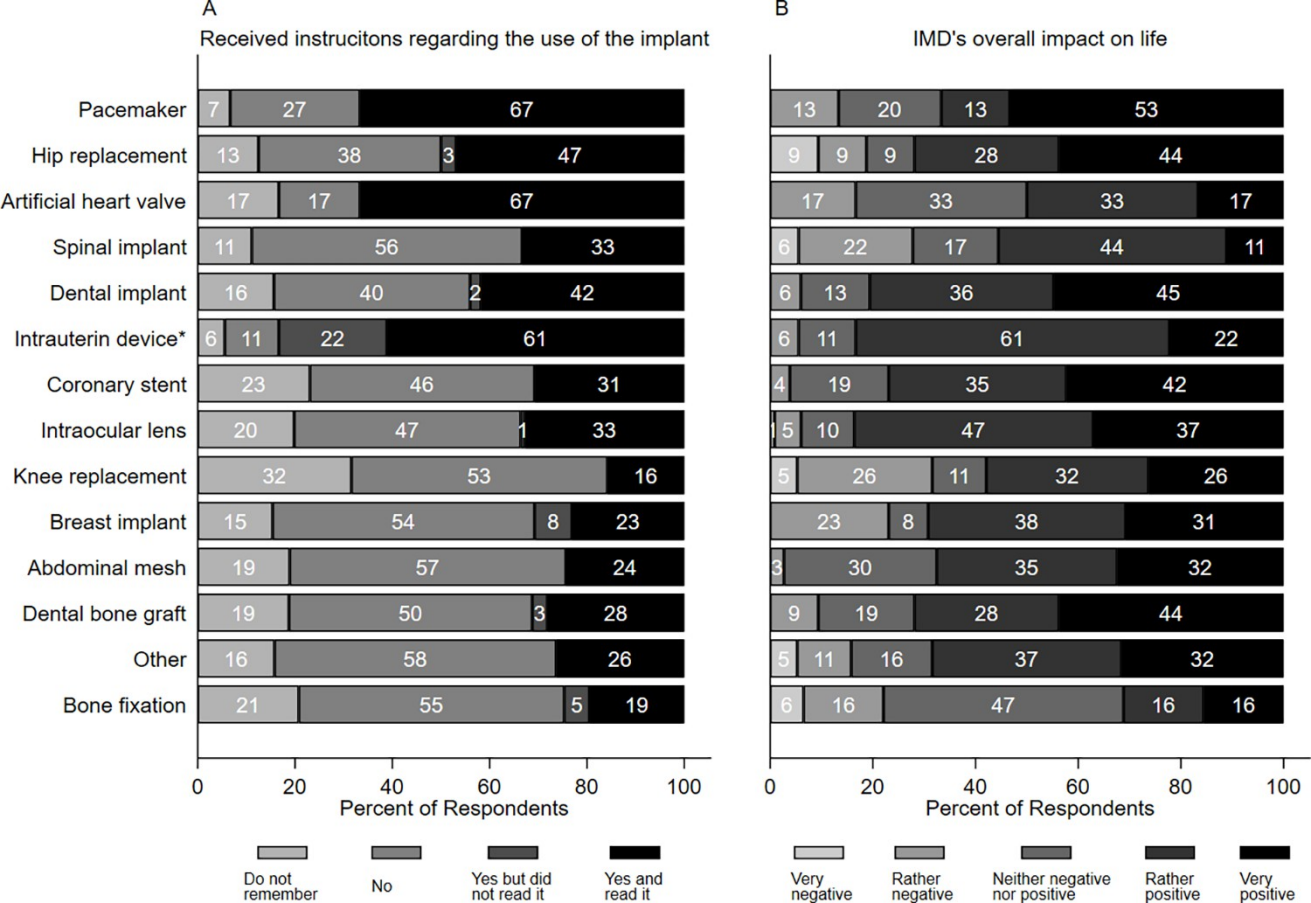
Share of responses on the received instructions for use for the IMD question and patients’ views about IMD’s overall impact on their life by IMD type (N = 433). *Only for women; Data for ‘glucose sensor’ are not presented as only one women case was identified.

In the subsample living with IMD, 46.2% reported that they did not get instructions for use for the IMD, and 17.3% were not able to recall whether instructions for use were provided or not. One third of respondents received instructions for use and read it (33.5%), and only very few (3.0%) did not read it. Instructions for use most often were given to patients who were wearing intrauterin device (83%), artificial heart valve (67%) and pacemaker (67%), while the proportion of those who reported not having received instructions for use was the highest for knee replacement (85%), abdominal mesh (76%) and bone fixation (76%). The share of respondents with or without instructions for use by IMD type is presented in Fig 2A. The duration of living with IMD by response categories on received instructions for use (S1 Table) significantly differed only for intrauterin device (ANOVA F_3,14_ = 13.66; p<0.01).

**Fig 3:**
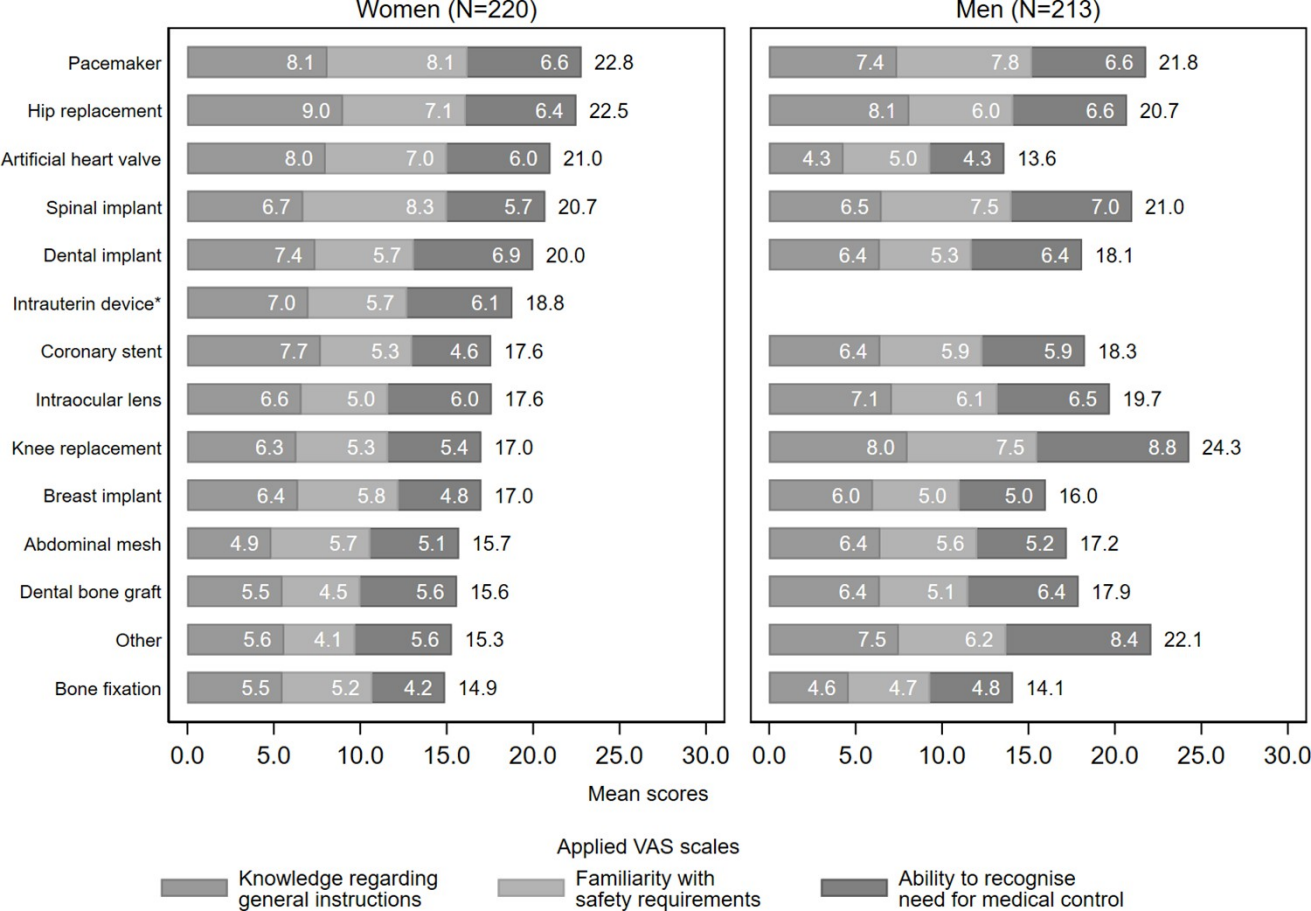
Patients’ knowledge regarding living with implantable medical devices by IMD type as measured on visual analogue scales (0 –‘not at all’; 10 –‘completely’). *Only for women; Numbers outside of columns represent the sum of the corresponding VAS scales. Results for ‘Ability to recognise need for information security or privacy control’ are not shown as it was reported only by patients wearing electronic devices. Data for ‘glucose sensor’ are not presented as only one women case was identified.

The average self-perceived familiarity with general instructions, safety requirements and ability to recognise need for medical control and information security or privacy control varied between 6.5 and 5.9 (as measured on a 0–10 VAS scale). Patients’ knowledge differed by sociodemographic variables, IMD’s overall impact on life and received instructions for use categories. Results are summarised in Table 3. Patients’ knowledge by IMD type is shown in Fig 3.

**Table 3:** Patients’ knowledge regarding living with implantable medical device (N = 433).

	Self-perceived familiarity with specific instructions and lifestyle advice for the daily use, VAS (0–10)*	Self-perceived familiarity with safety requirements, VAS (0–10)*	Self-perceived ability to recognise need for medical control, VAS (0–10)*	Self-perceived ablity to recognise need for information security or privacy control, VAS (0–10)*, N = 16**
**Total Mean (SD)**	6.5 (3.3)	5.5 (3.8)	5.9 (3.4)	5.9 (2.6)
**Sex**	p = 0.2434	p = 0.8783	p = 0.8455	p = 0.0074
*Men*	6.3 (3.3)	5.4 (3.8)	6.0 (3.2)	7.5 (2.5)
*Women*	6.7 (3.3)	5.5 (3.7)	5.8 (3.6)	4.3 (1.4)
**Age**	p = 0.0028	p = 0.0166	p = 0.1182	p = 0.7859
*40–44*	5.3 (3.5)	4.6 (3.8)	4.9 (3.5)	4.3 (3.1)
*45–49*	5.9 (3.3)	5.3 (3.7)	6.1 (3.3)	6.0 (2.8)
*50–54*	6.2 (3.1)	5.1 (3.4)	5.7 (3.4)	6.0 (-)
*55–59*	6.8 (3.0)	5.3 (3.4)	5.9 (3.2)	4.0 (-)
*60–64*	6.2 (3.3)	4.9 (4.0)	5.5 (3.4)	10.0 (-)
*65–69*	6.6 (3.2)	5.2 (3.7)	5.9 (3.3)	5.0 (-)
*70–74*	7.8 (3.0)	6.9 (3.5)	6.9 (3.2)	6.3 (3.5)
*75+*	6.8 (3.5)	6.1 (4.1)	6.1 (3.7)	6.3 (2.5)
**Education**	p = 0.0064	p = 0.0139	p = 0.0043	p = 0.4591
*Primary*	6.2 (3.0)	4.9 (3.6)	5.3 (3.4)	5.2 (3.5)
*Secondary*	6.1 (3.5)	5.1 (3.8)	5.5 (3.5)	5.4 (1.4)
*Tertiary*	7.1 (3.2)	6.1 (3.7)	6.6 (3.2)	7.5 (2.9)
**Settlement type**	p = 0.2186	p = 0.0509	p = 0.0456	p = 0.2961
*Capital*	6.9 (3.2)	6.2 (3.7)	6.5 (3.2)	6.6 (2.4)
*Town*	6.4 (3.3)	5.2 (3.7)	5.8 (3.4)	6.7 (2.7)
*Village*	6.1 (3.4)	5.1 (3.8)	5.2 (3.6)	4.2 (2.3)
**Married/having a partner**	p = 0.8700	p = 0.8652	p = 0.3840	p = 0.2145
*Yes*	6.5 (3.3)	5.4 (3.8)	5.8 (3.4)	6.8 (2.8)
*No*	6.5 (3.3)	5.5 (3.8)	6.0 (3.4)	4.7 (1.9)
**Living with someone in the household**	p = 0.8638	p = 0.8128	p = 0.6238	p = 0.7727
*Yes*	6.5 (3.3)	5.4 (3.7)	5.9 (3.4)	6.1 (3.0)
*No*	6.5 (3.3)	5.5 (3.8)	5.7 (3.5)	5.4 (1.1)
**Paid work**	p = 0.1885	p = 0.1623	p = 0.3422	p = 0.8529
*Yes*	6.4 (3.1)	5.2 (3.6)	5.8 (3.2)	6.0 (1.8)
*No*	6.6 (3.4)	5.6 (3.9)	5.9 (3.6)	5.8 (2.9)
**Houshold income category (per capita)** ^**b**^	p = 0.6274	p = 0.5159	**p = 0.0006**	p = 0.0415
*1st quintile*	6.3 (3.2)	5.5 (3.8)	5.5 (3.4)	3.8 (1.8)
*2nd quintile*	6.5 (3.3)	5.2 (3.6)	6.0 (3.5)	8.7 (2.3)
*3rd quintile*	6.5 (3.4)	6.0 (3.7)	5.7 (3.5)	6.3 (1.5)
*4th quintile*	6.3 (3.4)	4.9 (3.9)	5.0 (3.4)	4.5 (0.7)
*5th quintile*	6.9 (3.3)	5.7 (3.9)	7.1 (2.8)	10.0 (-)
**Received instructions for use for the implant**	**p = 0.0001**	**p = 0.0001**	**p = 0.0001**	p = 0.3062
Yes and read it	8.4 (2.0)	7.4 (2.9)	7.5 (2.5)	6.5 (2.5)
Yes but did not read it	5.5 (2.8)	4.5 (3.2)	4.4 (2.7)	- (-)
No	5.6 (3.5)	4.3 (3.8)	5.3 (3.5)	5.2 (2.7)
Do not remember	5.6 (3.2)	4.8 (3.6)	4.7 (3.6)	3.0 (-)
**IMD’s overall impact on life**	**p = 0.0001**	**p = 0.0001**	**p = 0.0001**	p = 0.1669
Very negative	5.0 (4.2)	3.7 (4.8)	4.4 (4.2)	- (-)
Rather negative	5.8 (3.2)	5.2 (3.5)	5.1 (3.4)	4.7 (3.5)
Neither negative nor positive	4.9 (3.2)	4.5 (3.3)	4.3 (3.2)	4.3 (1.5)
Rather positive	6.6 (2.9)	5.2 (3.6)	5.8 (3.2)	4.5 (0.7)
Very positive	7.7 (3.2)	6.4 (3.9)	7.2 (3.3)	7.3 (2.4)

p<0.001 significance level was applied to account for multiple hypothesis testing

*Questions were formulated as follows: ‘How familiar are you with the instructions and lifestyle advice for the daily use of the implanted device?’ (VAS, 0–10); ‘How familiar are you with the specific safety requirements for the implanted device you are using? (e.g. for airfield checks, medical imaging examinations, certain therapeutic procedures)’ (VAS, 0–10); ‘How confident are you that you will be able to recognise in time if there is a problem with the implanted device that needs medical control?’ (VAS, 0–10); ‘How confident are you that you will be able to recognise in time if there is a problem with the implanted device that requires information security or privacy control?’ (VAS, 0–10)

**Number of patients currently wearing electronic IMD

The knowledge scales were moderately correlated with SDM-Q-9, except recognising need for information security or privacy control, for which the correlation was weak (Note: This question was asked only in case of 16 patients, 15 with pacemaker and one with glucose sensor). Correlations were also found to be weak between EQ-5D-5L and all knowledge scales (S2 Table).

Significant but weak correlations were found between the knowledge VAS and eHEALS scores. (Note: correlations with EQ-5D-5L index, EQ VAS were also weak.) Results are summarized in S2 Table. The average eHEALS score was higher if patients have received instructions for use. Detailed results are shown in S1 Fig.

The correlations between knowledge VAS scales were strong or moderate (S2 Table). Cronbach’s alfa was 0.86 for the combined knowledge score, indicating good consistency. Numerical differences between men and women were observed by IMD type, however, regression analysis revealed that none of them were significant.

### IMD’s overall impact on patients’ life and its determinants

Among those who were living with IMD at the time of the survey, 10.9% patients reported that the IMD had very negative or rather negative impact on their life, 19.4% indicated that it was neither negative nor positive and 69.7% felt a rather positive or very positive effect. The share of patients who reported very or rather positive life impact was the greatest for intraocular lens (84%) and intrauterine device (83%). Patients’ views on the overall life impact of the IMD they are living with are shown on Fig 2B. Both very positive and very negative overall impact on life were associated with higher average eHEALS scores compared to other response options (S1 Fig).

In the multiple regression analysis, significant associations were found between several IMD types and IMD’s overall impact on life (model 1). Patients’ educational level (tertiary; model 2), health state (EQ-5D-5L index; model 2) and knowledge (model 3) were also significant determinants. The level of digital health literacy (eHEALS score) and if the patient has received instructions for use for the IMD and read it were not significantly associated with the IMD’s overall impact on life (models 4 and 5, respectively). Shared decision-making (SDM-Q-9), however, was found to be a significant determinant and cancelled the effect of patients’ knowledge (model 6) even if the model was controlled for all of the aforementioned variables (model 7). Results are summarized in S3 Table.

## Discussion

The aim of our study was to assess self-reported lifetime and point prevalence of IMDs, patients’ knowledge about living with IMD and to identify relevant factors affecting IMD’s overall impact on patients’ life. We have provided basic epidemiologic data on a broad spectrum of IMDs in the adult general population aged 40+ in Hungary. Substantial proportion (41.7%) of respondents have had an IMD, and about one third (30.9%) were still living with IMD at the time of the survey. Our findings suggest that a remarkable number of people is affected in the population by the MDR. Providing residents and patients with appropriate information is a major public health challenge. Although our study did not provide causal evidence, it seems that passive information sharing with patients is not sufficient, and involvement in shared decision-making is needed to achieve better self-percieved outcomes.

Expectations and attitude towards IMDs and knowledge of relevant interventions have been evaluated in previous studies. However, available data regarding patients’ IMD specific knowledge and its association with health outcomes are limited mainly to particular IMD types and comprehensive assessments across different device categories are still needed [4–7]. Klemetti et al. have demonstrated among patients undergoing hip and knee arthroplasty that expectations towards patient education were high, but there was a remarkable difference between expected and received knowledge. Their findings also highlight that a significant proportion of patients were not satisfied with the information they received [4]. The measurement tools used in our study were partly different as we investigated knowledge and information sharing specifically to IMDs, but our observations are consistent with these results. In our study, a great proportion of patients living with hip and knee implants reported that they had not received any instructions for use (38% and 53%, respectively). As we included a comprehensive set of IMDs, we had the opportunity to further investigate and compare how well patients were informed across different device categories. It was observed for several implant categories, that a similarly high rate of patients were not provided with instructions for use. It is important to mention that the difference in the duration of living with IMD by response categories on received instructions for use was only significant for one IMD type (intrauterin device) suggesting that the time since the implantation presumably does not have an influence on these responses. In addition, patients who received and read the IMD’s instructions for use reported significantly higher (better) knowledge scores suggesting that IMD specific knowledge may be related to how well patients are informed. However, the regression analysis revealed, that information sharing and knowledge were associated with IMD’s impact on life, but these associations have been overwritten by shared decision-making (SDM-Q-9), indicating that it may be a key factor for better health outcome. Patients who received instructions for use also had better eHEALS scores, which may indicate that they were more aware of how to utilize digital health information. However, the weak correlations found between eHEALS and knowledge VAS scores suggest, that better digital health literacy on its own does not necessarily result in higher level of patients’ knowledge about their IMDs.

Attitudes and knowledge regarding IMDs among patients living with cardiac implantable electronic devices have been studied in the literature. Haugaa et al found that a vast amount of respondents were sufficiently informed in general (44%), however many of them needed further information about possible complications and the functioning of their implant (i.e. capacity of the battery and physical activity limitations; 21% for each) [5]. Our findings are in line with these results as we also observed a very high overall knowledge level for patients wearing pacemaker compared to other IMDs.

It has also been reported in the study of Haugaa et al. that cardiac implantable electronic devices clearly or slightly improved the quality of life for 73% of patients but worsening was reported only for 5% (56% of patients had a pacemaker) [5]. The results of our study are consistent with this, as a comparable, albeit slightly lower proportion (63%) of patients with a pacemaker in our study reported that the device had a positive impact on their lives. We also found that positive life impact was reported to a great extent for several IMD types (i.e. intraocular lens, intrauterine device). However, for some IMD types, there were apparently higher rates of negative (i.e. spinal implant, knee replacement) and neutral impact (i.e. bone fixation). Also, those who felt that the IMD had very positive or rather positive overall impact on their lives had significantly higher average scores compared to those who experienced neutral or negative impact.

It has been described previously that patient education could have a considerable impact on outcomes of health care interventions. For instance, in patients who went through total knee or hip arthroplasty higher specific knowledge was associated with better functional outcomes [6, 7]. However, the relationship between patients’ knowledge and IMD’s overall impact onlife has remained unexplored so far. In our analysis, we observed this effect alongside other potential important determinants of IMDs’ impact on life. Patients’ average level of knowledge was associated with better life impact when controlled for IMD type, sociodemographic factors, duration since the first implant and health state. However, this effect was fully cancelled by SDM-Q-9, indicating that patient involvement in health care decision is a key factor and efficient communication plays an important role in delivering knowledge towards patients. In addition to this, patients’ digital health literacy and if they have received instructions for use were not associated with IMDs’ impact on life and these factors did not considerably alter the effect of knowledge. Furthermore, similarly high eHEALS scores were observed for patients with both very negative and very postitive life experience with IMDs, suggesting that even high level of digital health literacy can be associated with negative overall impact on life. These results suggest that setting up electronic information resources or providing information leaflets are not enough to properly inform patients about IMDs. It also suggests that solely passive information sharing (e.g., providing ‘Instructions for use’) is not sufficient to achieve positive life impact, but both physicians’ active contribution and patient involvement in health care decisions are essential. We believe that more attractive and usable communication channels have to be established between the device manufacturers and the patients. IMDs are becoming more-and-more intelligent. Hence in the near future, more-and-more patients have to be ready to receive important information about the recently identified vulnerabilities of the IMD, and also to let the device maintain regular integrity tests automatically and do security updates.

Some limitations of the study need to be mentioned. Firstly, respondents with secondary and tertiary education were slightly overrepresented. Such differences are commonly observed for online sampling methods, fairly similar results have been published before [12]. Therefore, the effect of the difference in educational level on our results is considered negligible. However, there were differences in sociodemographic variables (i.e. sex, age) by IMD type that may influence the results regarding the effect of the time since IMD implantation on whether patients could recall if instructions for use have received. Secondly, in some of the subgroups by IMD type, representativeness could not be ensured due to the low number of respondents, and therefore age and sex may differ from that observed in the general population. In addition to this, only few electronic devices (pacemaker and glucose sensor) were identified in our analysis that restricts the implication of the results for these categories. A further limitation is that although quota sampling is a popular and cost-effective method, it is not random and therefore may have a hidden bias. Moreover, we collected self-reported data directly from participants, and there were no medical confirmation in any cases. Also in relation to self-reports, recall bias can not be excluded in some instances, especially for questions concerning ever received IMD and received instructions for use of the implant. We did not explore in detail the type of written ‘Instructions for use’ (e.g., whether it was a manual provided by the manufacturer or it was an explanation included in the medical documentation or both) or how the instructions were handled to the patients. Investigating these details and patients’ preferences regarding the different modes of receiving instructions is a nice avenue for further research. A further limitation is that standardized tools to measure patients’ level of knowledge of IMDs are not available, therefore we applied simple knowledge VAS scales in our study. Although the associations described in our study can only be interpreted with the above detailed limitations, we believe that our results add to the scarce body of knowledge in the field of IMDs and can be used to generate future hypotheses for the scientific community.

High IMD prevalence and its expected increase in the future projects that significant amount of patients will be affected by the new MDR regulation which has to be applied to a wide range of IMDs. The results of our study raise the possibility that providing patients with the information sources (implant card) specified in the regulation may not always be sufficient to achieve the goals of patient safety and improved health outcomes, as involvement in decision-making and effective communication may be key factors in transferring knowledge to patients and improving their quality of life. Furthermore, our results provide basic epidemilogic data to be applied in planning social interventions aiming at vulnerable IMD patient groups and in organizing healthcare services.

## Conclusions

In conclusion, this study provides an estimate of the prevalence of IMDs in the 40+ years old Hungarian population. The results suggest that the proportion of patients living with IMDs is remarkable, indicating that the MDR presumably has a significant social impact. A substantial proportion of patients reported that have not received instructions for use of the device they are living with. The knowledge regarding their IMDs differs by the type of the device, patients having received instructions for use and those who have reported better impact on life experiences are generally more aware of their IMD. The findings of this study also suggest that patients’ information gathering may not be sufficient to achieve an appropriate outcome and the contribution of healthcare professionals to shared decision-making is an important factor, which, given the remarkable number of patients affected, is a major public health challenge. Therefore, it is recommended that the impact and cost-effectiveness of different strategies to inform patients should be investigated in future prospective studies.

## Supporting information

S1 Fig Distribution of eHEALS scores by received instructions for use and IMD’s overall impact on life. (TIF)

S1 Table Duration of living with IMD between responses on received instructions for use by IMD categories. Differences in duration of living with IMD between response categories by IMD type were analyzed with ANOVA test.
(DOCX)

S2 Table Spearman’s correlations in respondents currently living with IMD. *p<0.05; **p<0.01; ***p<0.001.
(DOCX)

S3 Table Observed effects on IMD’s overall impact on patients’ life in different regression models. *p<0.05; **p<0.01; ***p<0.001. ^a^All coefficients indicate multivariate analysis.
(DOCX)
