# Supplementary figures and images for: Epidemiology and patients’ self-reported knowledge of implantable medical devices: Results of a cross-sectional survey in Hungary
Source: PLoS One. 2023 Apr 18;18(4):e0284577. doi: 10.1371/journal.pone.0284577 (PMC10112797; doi:10.1371/journal.pone.0284577)

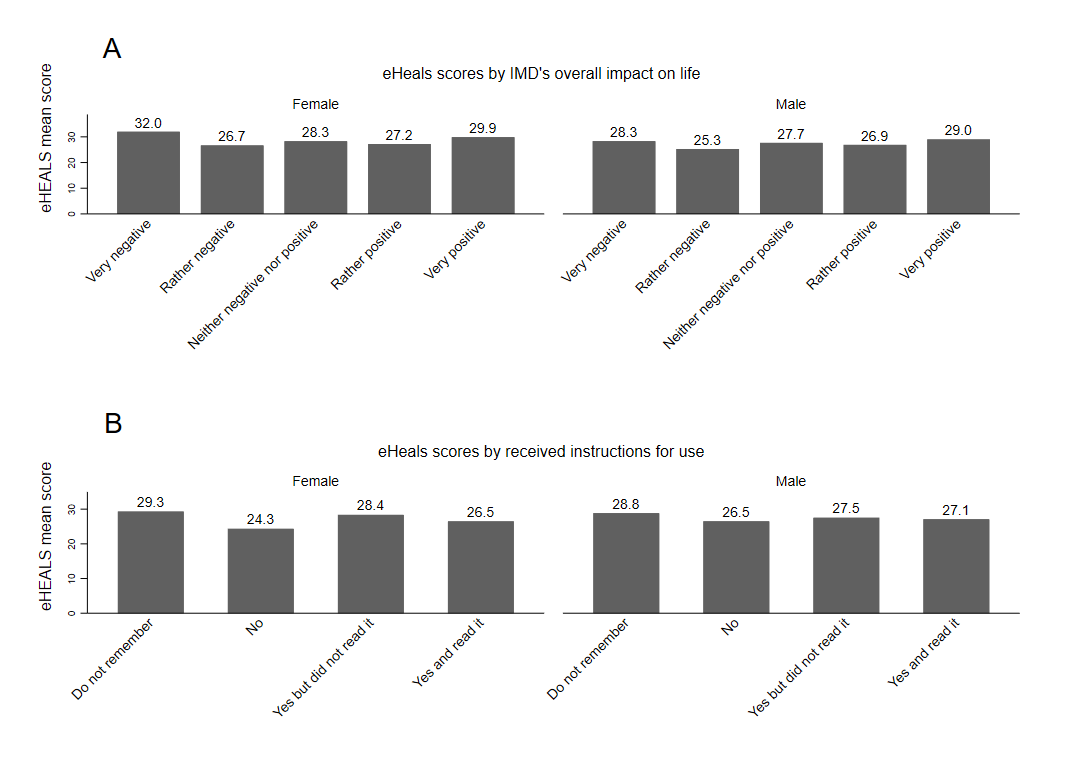

Supplement: S1 Fig — (TIF) [file pone.0284577.s001.tif]
