# Supplementary material for: Epidemiology and patients’ self-reported knowledge of implantable medical devices: Results of a cross-sectional survey in Hungary
Source: PLoS One. 2023 Apr 18;18(4):e0284577. doi: 10.1371/journal.pone.0284577 (PMC10112797; doi:10.1371/journal.pone.0284577)
Supplement: S1 Table — Differences in duration of living with IMD between response categories by IMD type were analyzed with ANOVA test. (DOCX) [file pone.0284577.s002.docx]

**S1 Table. Duration of living with IMD between responses on received instructions for use by IMD categories**

|  | | **Received instructions regarding the use of the implant** | | | |
| --- | --- | --- | --- | --- | --- |
|  |  | **Do not remember** | **No** | **Yes but did not read it** | **Yes and read it** |
| **Pacemaker** |  | p=0.49 | | | |
|  | mean years (SD) | 11.0 (0.0) | 12.3 (6.9) | NA | 7.2 (7.2) |
|  | N | 1 | 4 | NA | 10 |
| **Hip replacement** |  | p=0.34 | | | |
|  | mean years (SD) | 5.5 (5.3) | 11.3 (9.6) | 1.0 (0.0) | 7.3 (6.2) |
|  | N | 4 | 12 | 1 | 15 |
| **Artificial heart valve** |  | p=0.48 | | | |
|  | mean years (SD) | 4.0 (0.0) | 26.0 (0.0) | NA | 14.0 (11.4) |
|  | N | 1 | 1 | NA | 4 |
| **Spinal implant** |  | p=0.34 | | | |
|  | mean years (SD) | 13.5 (14.9) | 7.6 (6.9) | NA | 13.17 (7.3) |
|  | N | 2 | 10 | NA | 6 |
| **Dental implant** |  | p=0.19 | | | |
|  | mean years (SD) | 10.4 (10.1) | 9.0 (6.7) | 2.0 (2.7) | 7.3 (8.0) |
|  | N | 21 | 54 | 3 | 56 |
| **Intrauterin device** |  | **p<0.01** | | | |
|  | mean years (SD) | 45.0 (0.0) | 4.5 (0.7) | 7.5 (5.5) | 7.45 (6.2) |
|  | N | 1 | 2 | 4 | 11 |
| **Coronary stent** |  | p=0.61 | | | |
|  | mean years (SD) | 7.3 (7.6) | 6.7 (4.6) | NA | 9.3 (5.7) |
|  | N | 6 | 12 | NA | 8 |
| **Intraocular lens** |  | p=0.04 | | | |
|  | mean years (SD) | 10.5 (10.3) | 5.6 (5.1) | 9.0 (0.0) | 6.1 (6.7) |
|  | N | 23 | 54 | 1 | 38 |
| **Knee replacement** |  | p=0.15 | | | |
|  | mean years (SD) | 10.7 (8.1) | 6.5 (4.5) | NA | 2.7 (1.2) |
|  | N | 6 | 10 | NA | 3 |
| **Breast implant** |  | p=0.85 | | | |
|  | mean years (SD) | 12.5 (13.4) | 13.7 (12.5) | 3.0 (0.0) | 13.3 (5.7) |
|  | N | 2 | 7 | 1 | 3 |
| **Abdominal mesh** |  | p=0.75 | | | |
|  | mean years (SD) | 8.7 (7.5) | 8.8 (6.3) | NA | 7.0 (4.4) |
|  | N | 7 | 21 | NA | 9 |
| **Dental bone graft** |  | p=0.69 | | | |
|  | mean years (SD) | 7.2 (3.8) | 8.4 (7.4) | 5.0 (0.0) | 5.3 (5.4) |
|  | N | 6 | 16 | 1 | 9 |
| **Other** |  | p=0.44 | | | |
|  | mean years (SD) | 14.0 (8.9) | 12.2 (9.4) | NA | 6.8 (6.3) |
|  | N | 3 | 11 | NA | 5 |
| **Bone fixation** |  | p=0.35 | | | |
|  | mean years (SD) | 15.1 (14.5) | 10.0 (8.5) | 8.0 (9.8) | 12.8 (10.8) |
|  | N | 16 | 42 | 4 | 15 |
