# Supplementary material for: Epidemiology and patients’ self-reported knowledge of implantable medical devices: Results of a cross-sectional survey in Hungary
Source: PLoS One. 2023 Apr 18;18(4):e0284577. doi: 10.1371/journal.pone.0284577 (PMC10112797; doi:10.1371/journal.pone.0284577)
Supplement: S2 Table — *p<0.05; **p<0.01; ***p<0.001. (DOCX) [file pone.0284577.s003.docx]

**S2 Table. Spearman’s correlations in respondents currently living with IMD**

|  | **EQ-5D-5L index** | **EQ VAS** | **eHEALS** | **SDM-Q-9** | **Self-perceived familiarity with specific instructions and lifestyle advice for the daily use, VAS (0-10)** | **Self-perceived familiarity with safety requirements, VAS (0-10)** | **Self-perceived ability to recognise need for medical control, VAS (0-10)** | **Self-perceived ablity to recognise need for information security or privacy control, VAS (0-10)** |
| --- | --- | --- | --- | --- | --- | --- | --- | --- |
| **EQ-5D-5L index** | **1.00** |  |  |  |  |  |  |  |
| **EQ VAS** | 0.64*** | **1.00** |  |  |  |  |  |  |
| **eHEALS** | 0.10* | 0.16** | **1.00** |  |  |  |  |  |
| **SDM-Q-9** | 0.16*** | 0.22*** | 0.19*** | **1.00** |  |  |  |  |
| **Self-perceived familiarity with specific instructions and lifestyle advice for the daily use, VAS (0-10)** | 0.18*** | 0.22*** | 0.20*** | 0.50*** | **1.00** |  |  |  |
| **Self-perceived familiarity with safety requirements, VAS (0-10)** | 0.13** | 0.16** | 0.20*** | 0.45*** | 0.61*** | **1.00** |  |  |
| **Self-perceived ability to recognise need for medical control, VAS (0-10)** | 0.18*** | 0.19*** | 0.24*** | 0.48*** | 0.61*** | 0.62*** | **1.00** |  |
| **Self-perceived ability to recognise need for information security or privacy control, VAS (0-10)** | 0.13 | 0.21 | 0.47 | 0.23 | 0.48* | 0.58* | 0.32 | **1.00** |

***p<0.05; **p<0.01; ***p<0.001**
