# Supplementary material for: Epidemiology and patients’ self-reported knowledge of implantable medical devices: Results of a cross-sectional survey in Hungary
Source: PLoS One. 2023 Apr 18;18(4):e0284577. doi: 10.1371/journal.pone.0284577 (PMC10112797; doi:10.1371/journal.pone.0284577)
Supplement: S3 Table — *p<0.05; **p<0.01; ***p<0.001. aAll coefficients indicate multivariate analysis. (DOCX) [file pone.0284577.s004.docx]

**S3 Table. Observed effects on IMDs’ overall impact on patients’ life in different regression models.**

| **Variables** | **Model 1** | **Model 2** | **Model 3** | **Model 4** | **Model 5** | **Model 6** | **Model 7** |
| --- | --- | --- | --- | --- | --- | --- | --- |
|  | Coefficients^a^  (95% CI) | | | | | | |
| **Hip implant** | 0.197  (-0.167; 0.562) | 0.174  (-0.185; 0.533) | 0.067  (-0.292; 0.426) | 0.061  (-0.300; 0.421) | 0.049  (-0.310; 0.408) | -0.015  (-0.367; 0.336) | -0.027  (-0.381; 0.327) |
| **Knee implant** | -0.212  (-0.671; 0.246) | -0.162  (-0.605; 0.280) | -0.225  (-0.663; 0.213) | -0.225  (-0.664; 0.214) | -0.191  (-0.631; 0.248) | -0.309  (-0.738; 0.119) | -0.287  (-0.719; 0.145) |
| **Spinal implant** | -0.498*  (-0.962;  -0.033) | -0.189  (-0.651; 0.272) | -0.289  (-0.748; 0.169) | -0.292  (-0.751; 0.167) | -0.279  (-0.737; 0.178) | -0.286  (-0.733; 0.161) | -0.282  (-0.730; 0.166) |
| **Intraocular lens** | 0.410***  (0.177; 0.643) | 0.252  (0.000; 0.505) | 0.240  (-0.009; 0.489) | 0.239  (-0.011; 0.488) | 0.236  (-0.012; 0.485) | 0.206  (-0.037; 0.449) | 0.204  (-0.040; 0.448) |
| **Breast implant** | 0.069  (-0.489; 0.626) | -0.016  (-0.567; 0.534) | -0.030  (-0.573; 0.513) | -0.023  (-0.567; 0.522) | -0.011  (-0.554; 0.532) | -0.023  (-0.552; 0.506) | -0.008  (-0.540; 0.523) |
| **Tooth implant** | 0.539***  (0.303; 0.774) | 0.354**  (0.104; 0.603) | 0.304*  (0.056; 0.551) | 0.298*  (0.049; 0.547) | 0.289*  (0.041; 0.537) | 0.246*  (0.004; 0.489) | 0.236  (-0.008; 0.481) |
| **Dental bone graft** | 0.020  (-0.337; 0.376) | -0.076  (-0.420; 0.269) | -0.049  (-0.390; 0.291) | -0.054  (-0.395; 0.287) | -0.044  (-0.384; 0.296) | -0.034  (-0.366; 0.297) | -0.034  (-0.367; 0.299) |
| **Pacemaker** | 0.349  (-0.176; 0.875) | 0.412  (-0.092; 0.916) | 0.306  (-0.194; 0.806) | 0.300  (-0.202; 0.801) | 0.262  (-0.241; 0.764) | 0.338  (-0.150; 0.826) | 0.307  (-0.185; 0.800) |
| **Artificial heart valve** | -0.282  (-1.093; 0.528) | -0.415  (-1.196; 0.366) | -0.383  (-1.153; 0.387) | -0.387  (-1.158; 0.384) | -0.441  (-1.213; 0.332) | -0.505  (-1.258; 0.247) | -0.538  (-1.295; 0.218) |
| **Coronary stent** | 0.445*  (0.033; 0.855) | 0.349  (-0.059; 0.756) | 0.319  (-0.083; 0.721) | 0.320  (-0.082; 0.723) | 0.319  (-0.083; 0.720) | 0.235  (-0.158; 0.629) | 0.239  (-0.156; 0.633) |
| **Abdominal mesh** | 0.284  (-0.069; 0.637) | 0.186  (-0.166; 0.539) | 0.182  (-0.165; 0.529) | 0.179  (-0.169; 0.527) | 0.187  (-0.160; 0.533) | 0.209  (-0.130; 0.547) | 0.209  (-0.130; 0.548) |
| **Glucose sensor** | -2.180**  (-3.559;  -0.801) | -1.528*  (-2.870;  -0.185) | -1.481*  (-2.805;  -0.158) | -1.466*  (-2.793;  -0.140) | -1.437*  (-2.759;  -0.115) | -1.303*  (-2.595;  -0.011) | -1.274  (-2.570; 0.023) |
| **Other** | 0.064  (-0.383; 0.511) | -0.075  (-0.597; 0.446) | -0.077  (-0.591; 0.437) | -0.077  (-0.592; 0.437) | -0.068  (-0.582; 0.445) | 0.018  (-0.484; 0.521) | 0.021  (-0.482; 0.525) |
| **Bone fixation** | -0.356**  (-0.591;  -0.120) | -0.440***  (-0.692;  -0.189) | -0.410**  (-0.659;  -0.162) | -0.412**  (-0.660;  -0.163) | -0.403***  (-0.651;  -0.154) | -0.338**  (-0.583;  -0.094) | -0.336**  (-0.581;  -0.091) |
| **Intrauterin device** | 0.193  (-0.097; 0.483) | -0.016  (-0.375; 0.343) | -0.063  (-0.417; 0.292) | -0.070  (-0.426; 0.287) | -0.093  (-0.449; 0.263) | -0.063  (-0.409; 0.283) | -0.085  (-0.435; 0.265) |
| **Sex** |  |  |  |  |  |  |  |
| **Male** |  | 0.031  (-0.169; 0.232) | 0.025  (-0.172; 0.223) | 0.026  (-0.172; 0.223) | 0.036  (-0.162; 0.233) | 0.023  (-0.170; 0.215) | 0.029  (-0.164; 0.223) |
| **Age** |  | 0.005  (-0.005; 0.014) | 0.003  (-0.006; 0.012) | 0.003  (-0.006; 0.012) | 0.003  (-0.006; 0.012) | 0.003  (-0.005; 0.012) | 0.004  (-0.005; 0.013) |
| **Education** |  |  |  |  |  |  |  |
| **Secondary** |  | 0.198  (-0.045; 0.441) | 0.213  (-0.027; 0.453) | 0.211  (-0.030; 0.451) | 0.223  (-0.016; 0.463) | 0.189  (-0.045; 0.423) | 0.195  (-0.040; 0.430) |
| **Tertiary** |  | 0.330*  (0.064; 0.596) | 0.311*  (0.049; 0.574) | 0.308*  (0.045; 0.571) | 0.329*  (0.066; 0.592) | 0.323*  (0.067; 0.578) | 0.331*  (0.074; 0.589) |
| **Residency** |  |  |  |  |  |  |  |
| **City** |  | 0.095  (-0.120; 0.309) | 0.126  (-0.086; 0.338) | 0.125  (-0.087; 0.337) | 0.117  (-0.095; 0.329) | 0.114  (-0.092; 0.321) | 0.108  (-0.099; 0.316) |
| **Village** |  | -0.012  (-0.295; 0.270) | 0.023  (-0.256; 0.302) | 0.026  (-0.254; 0.305) | 0.030  (-0.249; 0.308) | 0.045  (-0.227; 0.317) | 0.050  (-0.223; 0.323) |
| **Duration since first implant** |  | 0.009  (-0.001; 0.019) | 0.009  (-0.001; 0.019) | 0.009  (-0.001; 0.019) | 0.010  (0.000; 0.019) | 0.008  (-0.001; 0.018) | 0.009  (-0.001; 0.018) |
| **EQ-5D-5L index score** |  | 0.813***  (0.470; 1.157) | 0.720***  (0.378; 1.062) | 0.719***  (0.377; 1.062) | 0.718***  (0.376; 1.059) | 0.662***  (0.328; 0.997) | 0.662***  (0.328; 0.997) |
| **Combined knowledge VAS score** |  |  | 0.057***  (0.026; 0.088) | 0.056***  (0.024; 0.087) | 0.046**  (0.012; 0.080) | 0.020  (-0.014; 0.054) | 0.014  (-0.023; 0.050) |
| **eHEALS score** |  |  |  | 0.004  (-0.013; 0.020) |  |  | 0.002  (-0.015; 0.018) |
| **Received instructions for use and read it** |  |  |  |  | 0.166  (-0.048; 0.379) |  | 0.100  (-0.111; 0.311) |
| **SDM-Q-9 score** |  |  |  |  |  | 0.021***  (0.012; 0.029) | 0.020***  (0.011; 0.029) |
| **Observations** | 433 | 426 | 426 | 426 | 426 | 426 | 426 |
| **Constant** | 3.651^***^  (3.420; 3.882) | 2.507^***^  (1.844; 3.171) | 2.363^***^  (1.705; 3.022) | 2.271^***^  (1.481; 3.062) | 2.343^***^  (1.685; 3.001) | 1.974^***^  (1.312; 2.637) | 1.929^***^  (1.144; 2.713) |
| **R^2^** | **0.151** | **0.237** | **0.261** | **0.261** | **0.265** | **0.299** | **0.301** |

***p<0.05; **p<0.01; ***p<0.001**

**^a^All coefficients indicate multivariate analysis**
